# Public knowledge of risk factors and warning signs for cardiovascular disease among young and middle-aged adults in rural Tanzania

**DOI:** 10.1186/s12889-020-09956-z

**Published:** 2020-11-30

**Authors:** Alfa J. Muhihi, Amani Anaeli, Rose N. M. Mpembeni, Bruno F. Sunguya, Germana Leyna, Deodatus Kakoko, Anna Tengia Kessy, Mary Mwanyika Sando, Marina Njelekela, David P. Urassa

**Affiliations:** 1 Department of Community Health, Muhimbili University of Health and Allied Sciences, United Nations Road, P. O. Box 65001, Upanga, Dar es Salaam, Tanzania; 2 Africa Academy for Public Health, Plot # 802, Mwai Kibaki Road, Mikocheni, Dar es Salaam, Tanzania; 3 Lown Scholars Program, Department of Global Health and Population, Harvard T.H. Chan School of Public Health, Boston, MA USA; 4 Department of Development Studies, Muhimbili University of Health and Allied Sciences, United Nations Road, Upanga, Dar es Salaam, Tanzania; 5 Department of Epidemiology and Biostatistics, Muhimbili University of Health and Allied Sciences, United Nations Road, Upanga, Dar es Salaam, Tanzania; 6 Department of Behavioral Sciences, Muhimbili University of Health and Allied Sciences, United Nations Road, Upanga, Dar es Salaam, Tanzania; 7 Department of Physiology, Muhimbili University of Health and Allied Sciences, United Nations Road, Upanga, Dar es Salaam, Tanzania; 8 Deloitte Consulting Limited, Aris House, Plot # 152, Haile Selassie Road, Oysterbay, Dar es Salaam, Tanzania

**Keywords:** Knowledge, Cardiovascular diseases, Risk factors, Warning signs, Tanzania

## Abstract

**Background:**

Improving cardiovascular health requires public knowledge and reduction of modifiable cardiovascular disease (CVD) risk factors. This study assessed knowledge of risk factors and warning signs for CVDs among young and middle-aged adults in Morogoro, Tanzania.

**Methods:**

We conducted a community-based survey as part of cluster randomized controlled study of community health workers (CHWs) intervention for reduction of blood pressure among young and middle-aged adults in rural Morogoro. Information on socio-demographic characteristics, knowledge of risk factors and warning signs for CVDs was collected using an interviewer administered questionaire. Knowledge was assessed using open-ended questions followed by closed-ended questions. Descriptive statistics were used to describe knowledge of risk factors and warning signs. Logistic regression analysis was used to investigate factors associated with adequate knowledge of risk factors and warning signs for CVDs.

**Results:**

Two-thirds (65.7%) of the participants had heard about CVDs. The main sources of information were mainly relatives/ neighbors (64.8%) and radio (53.0%). Only 28.3% of the participants reported health care providers as source of information about CVDs. More than half of the participants (52.4%) did not mention even one risk factor spontaneously while 55.2% were unable to mention any warning sign. When asked to select from a list, 6.9% were unable to correctly identify any risk factor whereas 11.8% could not correctly identify even a single warning sign. Quarter of participants (25.4%) had good knowledge score of risk factors, 17.5% had good knowledge score of warning signs and 16.3% had overall good knowledge of both risk factors and warning signs. Residing in Ulanga, having higher education level, having ever checked blood pressure and being overweight/obese predicted adequacy of knowledge score for both risk factors and warning signs.

**Conclusion:**

Knowledge of risk factors and warning signs in this rural population of young and middle-aged adults was generally low. Health care providers were less likely to provide health education regarding risk factors and warning signs for CVDs. Health promotion interventions to increase population knowledge of risk factors and warning signs should be implemented for successful reduction of CVDs in Tanzania.

## Background

Cardiovascular Diseases (CVDs), a group of disorders that affect the heart and/or blood vessels are the leading causes of deaths and disability-adjusted life-years (DALYs) globally [1, 2]. In 2017, CVDs accounted for an estimated 17.8 million deaths, with more than 80% occurring in low- and middle-income countries (LMICs) [2]. It is projected that by the year 2030, CVDs will cause more than 23.6 million deaths [3] with stroke and coronary heart diseases being the main contributors. The rising burden of CVDs in LMICs is fueled by higher prevalence of risk factors such as smoking, physical inactivity, unhealthy dietary habits, overweight and obesity [4]. Population level reduction in risk factors such as blood pressure, total cholesterol, smoking and physical inactivity contributed to significant decline in CVD-related morbidity and mortality in high income countries [5–7].

A crucial step towards reduction in CVDs and improvement in cardiovascular health requires public knowledge of risk factors. Studies have shown that knowledge of CVDs and their risk factors can lead to success in their prevention and control [8] by influencing individual attitude and practices towards healthy lifestyle [9], improved compliance with treatment and decreased risk of disease complications [10]. The world health organization (WHO) promotes knowledge of behavioral CVD risk factors [11]. Already, some studies have assessed knowledge of CVD and risk factors in Africa among the general community [12–17], while a few others have done so in specific population groups [18–24].

Knowledge of risk factors for CVDs in sub-Saharan Africa (SSA) is generally poor. Findings from a systematic review of studies conducted in SSA indicated low levels of knowledge coupled with unfavorable perception towards CVD risk [25]. Another report from Uganda found that up to three-quarter of the surveyed population were unable to identify even a single risk factor or clinical symptom for CVD [16]. Understanding of the community about CVDs has significant positive implications as it helps development of targeted health education and promotion programs for prevention and control of CVDs [26].

Despite the rising prevalence CVDs and their related risk factors in Tanzania [27, 28], there are no published studies to date that have comprehensively assessed knowledge of CVDs and their risk factors in the Tanzanian general population. Understanding knowledge, perception and practices is crucial to inform the design and implementation of context appropriate intervention for prevention of the rising burden of CVDs in Tanzania. This study assessed knowledge of risk factors and warning signs for CVD among young and middle-aged adults in rural Tanzanian and further explored factors associated with knowledge.

## Methods

### Study design and setting

The study design and data collection methods have already been described elsewhere [29]. A baseline survey was conducted between 15th July and 30th August, 2019 as part of a cluster randomized controlled trial of Community Health Workers (CHWs) interventions for reduction of blood pressure among adults aged 25–64 years in Kilombero and Ulanga districts. The study area is approximately 450 km by road South-West of the Tanzania’s commercial capital of Dar es salaam. Kilombero and Ulanga districts cover a total surface area of 28,669 km^2^. Based on the 2012 national census and the average annual population increase for Morogoro region [30], Kilombero and Ulanga districts were projected to have a total population of 659,810 by end of 2019. The predominant tribes in Kilombero and Ulanga districts are Wapogolo and Wandamba with the main economic activities being maize and rice farming as well as fishing activities on the Kilombero river.

The baseline survey was conducted in 12 randomly selected villages (6 from Kilombero district and another 6 from Ulanga district) in Morogoro region. Study villages were selected from a list of 38 villages which had active CHWs at the time of randomization. For the CHWs intervention study, three villages from each district were assigned to intervention arm and the remaining three to control arm. Conditional randomization was done to ensure equal number of villages are assigned to intervention and control in each district. Based on the local government registries, the number of households per village ranged from 1587 to 5929 and a total of 262 households were randomly selected from each of the 12 study villages.

### Study participants and eligibility criteria

The study population included young and middle-aged adults 25–64 years, who resided in the selected villages and provided a written informed consent to participate. A resident was defined based on the Health and Demographic Surveillance System (HDSS) definition as an individual who has stayed in the selected household for at least 4 months continuously regardless of whether s/he had slept in that household a night before the interview [31]. A household was defined as group of people who served food from the same pot.

### Sample size estimation and sampling procedures

The sample size was estimated according to the methods proposed in the WHO stepwise approach to chronic disease risk factors surveillance (STEPS) [32]. The sample size was calculated for 95% CI (z = 1.96) on the basis of a 5% margin of error and an estimated prior population prevalence of hypertension of 25.9% from the national wide representative STEPS survey in Tanzania [33], a presumed design effect of 1.2 and an anticipated nonresponse rate of 10%. Participants were adults aged 25–64 years and categorized into four age-sex groups, resulting into 8 strata. The resulting minimum sample size for the study was 3145.

We used multi-stage cluster sampling technique with villages as clusters. The villages were stratified by district, to obtain equal number of villages in both districts. Furthermore, a random sample of 262 households was drawn for all villages and at each selected household, one eligible respondent was selected for interview. The next birthday rule was used to select one eligible respondent from a household where the research assistants first noted the months of birth of all eligible individuals in a household in ascending order (January – December). The first person on the list was then selected for interview, and if the selected person was not available after two attempts, then the next person on the list was interviewed. Non-response was considered if an eligible participant refused/did not consent to participate or the selected household had only one eligible responded who could not be found after two follow up attempts.

### Data collection procedures

Face-to-face interviews were conducted by a team of trained research assistants with experience in conducting health and demographic surveys. A modified WHO STEPs questionnaire was used for data collection (supplementary file). Information collected during the interviews included socio-demographic and economic characteristics, knowledge of risk factors and warning signs for cardiovascular diseases, medical history, behavioral characteristics and physical measurements.

#### Socio-demographic and economic characteristics

Socio-demographic information included age, gender, marital status, education level, and occupation. Age was collected as a continuous variable and categorized into 25–34, 35–44, 45–54, and 55–64 years. Education level was measured by asking the highest education level attained as none, primary education, secondary education, college/university. Marital status at the time of data collection was grouped into three groups as never married, married or living together and divorced /separated /widowed. Occupation was assessed and categorized as farmer, housewife, employed (public/private), petty business and others. Economic status of the participants was assessed through ownership of household items such as radio, television, telephone, sofa, refrigerator, bicycle, car, and having working electricity; house ownership, construction materials (floor, walls and roofing materials); source of fuel for cooking and lighting; source of water supply for home use and drinking; and household sanitation facility [34]. All this information was used to generate household wealth index following descriptions in the Demographic Health Survey (DHS) toolkit [35].

#### Assessment of CVD knowledge

To assess general knowledge, participants were asked if they had ever heard or read about CVDs and their sources of information. Knowledge of risk factors and warning signs was then assessed using open-ended questions (participants were asked to mention risk factors and warning signs spontaneously) followed by closed-ended questions (participants were asked to identify risk factors and warning signs from a list of “yes/no” questions). The questionnaire comprised of 10-items about knowledge of risk factors and 9-items on knowledge of warning signs. Knowledge scores were assigned as 1 (one) for each correct response and 0 (zero) for wrong response. Knowledge scores were calculated based on closed-ended questions.

The score points were then summed across to obtain a total score for knowledge of risk factors and warning signs separately. Total scores for knowledge of risk factors were categorized as; good (7–10 points), moderate (4–6 points), poor knowledge (1–3 points) and not knowledgeable at all (0 point). Total scores for knowledge of warning signs were categorized as; good (7–9 points), moderate (4–6 points) and poor knowledge (1–3 points) and not knowledgeable at all (0 point). Internal consistency and reliability of each set of items for assessment of knowledge of risk factors and warning signs was evaluated using Cronbach’s alpha test. The reliability coefficients were 0.826 for the scale of knowledge of risk factors and 0.782 for the scale of knowledge of warning signs. The two scales were reliable as Robinson and colleagues assert, an alpha coefficient above 0.80 is “exemplary”, and that in the range between 0.70 and 0.79 is “extensive” [36].

Total knowledge of CVDs was assessed by combining the total knowledge scores for risk factors and warning signs. The maximum possible total knowledge score was 19 points. Participants who obtained total knowledge score of ≥14 points were classified as having “good knowledge”, those with total knowledge score between 8 and 13 points were classified as having “moderate knowledge”, those with total knowledge score between 1 and 7 points were classified as having “poor knowledge”, and those who did not get any score as “not knowledgeable”.

#### Medical history and behavioral risk factors

Participants were asked if they had ever been diagnosed with hypertension or diabetes mellitus. Lifestyle related CVD risk factors including smoking, alcohol drinking, and dietary habits were assessed using a modified WHO STEPs questionnaire (supplementary file) which has been previously used in Tanzania [33]. Questions on smoking probed current and past smoking while questions for alcohol probed current and past drinking. Dietary assessment included frequency of consumption of fruits and vegetables (in days per week), and frequency of use of raw table salt (as never, sometimes or always).

#### Perceptions about CVD and practices towards CVD prevention

The study questionnaire also contained a section with set of questions that assessed participant’s self-perceived risk for cardiovascular disease, perception about current body weight, general practices, dietary practices, and behavioral practices in the past 1 year that relate to better cardiovascular health and prevention of CVDs.

#### Physical measurements

Blood pressure was measured using a digital blood pressure machine (OMRON HEM-712C, Omron Healthcare, Inc). Research assistants were trained to measured blood pressure on the left arm with participant in a seated position. The first reading was taken after at least 5 min of resting. The second and third readings were taken half-way and at the end of interview respectively. Participants who had elevated blood pressure had blood pressure measurements repeated on the following day to confirm their elevated blood pressure before being given referral letter to nearby health facility for proper diagnosis and management per standing national guidelines. For these individuals, we used their repeat blood pressure measurements for analysis. An average of three blood pressure measurements was used during analysis. Hypertension was defined as average systolic blood pressure (SBP) ≥140 mmHg and/or diastolic blood pressure (DBP) ≥90 mmHg and/or currently taking blood pressure lowering medications in accordance with the Seventh Report of the Joint National Committee on Prevention, Detection, Evaluation and Treatment of High Blood Pressure [37]. Anthropometric measurements included weight and height. Body weight was measured to the nearest 0.1 kg using a SECA 803 digital scale placed on flat ground with participant wearing light clothing and with no shoes. Height was measured to the nearest 0.1 cm with participant in a standing position with heels perpendicular to the portable stadiometer. Body mass index (BMI) was calculated using measured weight and height (kg/m^2^).

### Data analysis

Data were entered and analysed using IBM-Statistical Package for Social Sciences (SPSS Inc., Chicago, IL, USA) version 20 software for Windows. Descriptive statistics were used to summarize continuous variables as means with standard deviations and categorical variables as frequencies with proportions. Comparisons between groups were done using Chi-square test and independent samples t-test (or ANOVA) for categorical and continuous variables respectively.

To perform logistic regression, knowledge of CVD risk factors and warning signs was first dichotomized by combining good knowledge and moderate knowledge as having “adequate knowledge” and those with poor and not knowledgeable as having “inadequate knowledge”. Bivariate logistic regression was then performed to determine the relationship of each independent variable with CVD knowledge score. Predictor variables were categorized as: sex, age, wealth status, marital status, education level, place of residence, occupation, current smoker, current alcohol drinker, use of raw table salt, fruits consumption per week, vegetable consumption and body mass index.

All variables with *p* ≤ 0.2 in the bivariate analysis were included in the multiple logistic regression analysis to determine factors that are independently associated with overall adequate CVD knowledge. Before fitting multiple logistic regression model, we first checked for correlation and strength of correlation between variables in the regression model to avoid problem with multicollinearity. We used variance inflation factor (VIF) with a cut-off value of ≤5 to rule out multicollinearity. Overall fitness of the model was assessed using the Pearson Chi-square test and Hosmer–Lemeshow goodness-of-fit test. Adjusted odds ratio (AOR) with their corresponding 95% confidence intervals (95% CI) are presented as measures of association. Statistical significance was accepted based on two-sided *p*-value ≤0.05.

## Results

### General characteristics of study participants

A total of 3000 participants had complete data for analysis, giving a response rate of 95.4%. General characteristics of the study population are described in Table 1. The median age [interquartile range (IQR)] of the participants was 39 [31-48] years. Majority of the participants were women (74.1%), were married (71.5%), had primary education (80.4%) and were predominantly farmers (92.5%). With regard to behavioral and physiological characteristics, 5.9 and 19.7% of the participants were current smokers and alcohol drinkers respectively. Most participants (85.2%) used table salt, with 6.8% reporting to do so always. Less than two-thirds (63.9%) reported to consume vegetables 5–7 days per week while only 7.9% consumed fruits 5–7 days per week. More than quarter of participants (29.3%) were hypertensive, 28.5% were overweight (28.5%) and 16.3% were obese.

**Table 1:** **Characteristics of the study participants**

Variable	n (%) or median (IQR)
Age (Years) [Median (IQR)]	39 (31.0–48.0)
Age group (Years)	
25–34	1072 (35.7)
35–44	888 (29.6)
45–54	628 (20.9)
55–64	412 (13.7)
Sex	
Males	778 (25.9)
Females	2222 (74.1)
District of residence	
Kilombero	1500 (50.0)
Ulanga	1500 (50.0)
Marital status	
Never married	383 (12.8)
Married	2144 (71.5)
Separated/Divorced/Widowed	473 (15.8)
Education level	
No formal education	297 (9.9)
Primary education	2411 (80.4)
Secondary education	255 (8.5)
College/university education	37 (1.2)
Occupation	
Farmer	2775 (92.5)
House wife	79 (2.6)
Employed	27 (0.9)
Petty business	80 (2.7)
Other^a^	39 (1.3)
Current smoking status	
Yes	176 (5.9)
No	2824 (94.1)
Current alcohol drinking status	
Yes	591 (19.7)
No	2409 (80.3)
History of Diabetes Mellitus	
Yes	29 (1.0)
No	2971 (99.0)
Hypertensive	
Yes	880 (29.3)
No	2120 (70.7)
Body Mass Index [BMI (kg/m^2^)]	
Normal	1563 (52.1)
Overweight	855 (28.5)
Obese	489 (16.3)
Underweight	93 (3.1)

^**a**^Other occupations included: retired (0.3%), student (0.1%), no job (0.5%) and declined to respond (0.4%)

### General knowledge about CVDs

As presented in Table 2, about two-thirds (65.7%) of the participants had heard or read about CVDs. The main sources of information were relatives/neighbors (64.8%) and radio (53.0%). Only 28.3% of the participants reported health care providers as source of information about CVDs. There were significant gender differences in the sources of information about CVDs with radio and television being most common sources of information among men (all *p* < 0.001). On the contrary, higher proportion of women reported to have heard about CVDs from health care providers and relatives/neighbors (all *p* < 0.001).

**Table 2:** Awareness and source of information about CVDs

Knowledge about CVDs	All (***N*** = 3000)	Men (***N*** = 778)	Women (***N*** = 2222)	***P***-value
	Yes, n (%)	Yes, n (%)	Yes, n (%)	
Ever heard or read about CVDs				
Yes	1971 (65.7)	526 (67.6)	1445 (65.7)	0.192
No	1029 (34.3)	252 (32.4)	777 (35.0)	
Source of information				
Radio	1045 (53.0)	327 (62.2)	718 (53.0)	< 0.001
Television	164 (8.3)	64 (12.2)	100 (6.9)	< 0.001
Newspapers	22 (1.1)	13 (2.5)	9 (0.6)	0.001
Health care providers	558 (28.3)	100 (19.0)	458 (31.7)	< 0.001
Relative/neighbor	1278 (64.8)	284 (54.0)	994 (68.8)	< 0.001
Internet/social media	20 (1.0)	9 (1.7)	11 (0.8)	0.063
Other sources	13 (0.7)	3 (0.6)	10 (0.7)	0.768

### Knowledge of risk factors and warning signs

Results on participant’s knowledge of risk factors are summarized in Table 3. Most participants were not knowledgeable about risk factors when asked open-ended questions. Stress (35.8%) and cholesterol (18.5%) were the frequently mentioned risk factors. On asking closed-ended questions most participants identified stress (85.4%), obesity (67.7%), cholesterol (64.3%) and physical inactivity (63.3%). The least identified risk factors were family history of stroke (18.4%) and alcohol drinking (25.5%). Although half of the participants (51.4%) were able to identify five or more risk factors, 207 (6.9%) were unable to identify any risk factor.

**Table 3:** Knowledge of risk factors for CVDs

	Number (%)
**Spontaneous mention of risk factors for CVDs (Open-ended questions)**	
Old age	82 (2.7)
Obesity	277 (9.2)
Hypertension	73 (2.4)
Diabetes mellitus	51 (1.7)
Cholesterol	556 (18.5)
Smoking	17 (0.6)
Alcohol drinking	34 (1.1)
Physical inactivity	241 (8.0)
Family history of stroke	7 (0.2)
Stress	1073 (35.8)
*Number of risk factors mentioned (out of 10)*	
None	1574 (52.4)
1–4	1415 (47.2)
5–9	11 (0.4)
**Selecting risk factors for CVDs from a list (Close-ended questions)**	
Old age	1061 (35.4)
Obesity	2031 (67.7)
Hypertension	1098 (36.6)
Diabetes mellitus	1141 (38.0)
Cholesterol	1928 (64.3)
Smoking	794 (26.5)
Alcohol drinking	765 (25.5)
Physical inactivity	1899 (63.3)
Family history of stroke	552 (18.4)
Stress	2561 (85.4)
*Number of risk factors identified (out of 10)*	
None	207 (6.9)
1–4	1251 (41.7)
5–9	1542 (51.4)

Table 4 summarizes results of knowledge of warning signs for CVDs. When asked open-ended questions, again most participants had poor knowledge of warning signs. Only 24.5 and 18.4% of the participants mentioned shortness of breath and headache respectively as warning signs. On closed-ended questions, warning signs correctly identified by most participants were shortness of breath (74.3%), loss of consciousness (63.7%), headache (60.8%) and dizziness (60.2%). Jaw or neck pain (10.6%) and vomiting (11.0%) were the least identified warning signs.

**Table 4:** Knowledge about warning signs for CVD event

	Number (%)
**Spontaneous mention of warning signs for CVD (Open-ended questions)**	
Severe headache	553 (18.4)
Chest pain	99 (3.3)
Shortness of breath	736 (24.5)
Sweating while at rest	122 (4.1)
Vomiting	15 (0.5)
Pain in the jaw or neck	16 (0.5)
Pain in the arms or shoulder	169 (5.6)
Loss of consciousness	289 (9.6)
Dizziness	280 (9.3)
*Number of warning signs mentioned (out of 9)*	
None	1657 (55.2)
1–4	1322 (44.1)
5–9	21 (0.7)
**Selecting warning signs for CVD from a list (Close-ended questions)**	
Severe headache	1824 (60.8)
Chest pain	1324 (44.1)
Shortness of breath	2230 (74.3)
Sweating while at rest	1265 (42.2)
Vomiting	330 (11.0)
Pain in the jaw or neck	318 (10.6)
Pain in the arms or shoulder	1299 (43.3)
Loss of consciousness	1910 (63.7)
Dizziness	1807 (60.2)
*Number of warning signs identified (out of 9)*	
None	354 (11.8)
1–4	1314 (43.8)
5–9	1332 (44.4)

Participants’ knowledge scores for risk factors, warning signs and both combined are presented in Table 5. At least one in four participants had good knowledge while 35.7% had either poor or no knowledge at all about risk factors. As for warning signs, only 17.5% of the participants had good knowledge of warning signs whereas 40.1% had either poor or no knowledge of warning signs. Overall, only 16.3% of the participants had good knowledge of both risk factors and warning signs while 42.8% had moderate knowledge. The remaining 40.9% had poor or were not knowledgeable at all about risk factors and warning signs for CVDs.

**Table 5:** Participant knowledge scores for risk factors and warning for CVD event

	Number (%)
**Knowledge scores on risk factors for CVDs**	
Good knowledge	762 (25.4)
Moderate knowledge	1167 (38.9)
Poor knowledge	862 (28.7)
Not knowledgeable	209 (7.0)
**Knowledge scores on warning signs for CVD event**	
Good knowledge	524 (17.5)
Moderate knowledge	1274 (42.5)
Poor knowledge	848 (28.3)
Not knowledgeable	354 (11.8)
**Overall knowledge scores for risk factors and warning signs**	
Good knowledge	488 (16.3)
Moderate knowledge	1285 (42.8)
Poor knowledge	1073 (35.8)
Not knowledgeable	154 (5.1)

### Predictors of adequate knowledge of risk factors and warning signs

Table 6 summarizes predictors of adequate knowledge of risk factors and warning signs for CVDs in the study population. Sociodemographic characteristics associated with adequate knowledge were older age, male sex, being married, higher education level and employment status (all *p* < 0.05). Middle aged participants had significantly higher knowledge of warning signs compared to their counterpart younger participants. Men had good knowledge of risk factors than women, though no significant differences were observed for knowledge of warning signs. Participants from Ulanga district had significantly higher knowledge compare to Kilombero district (*p* < 0.001). Participants who reported to smoke had adequate knowledge of risk factors and warning signs than those who did not (*p* = 0.014). As for medical history, participants with history of diabetes mellitus, ever checked blood pressure and those who were hypertensive had higher knowledge (all *p* < 0.05).

**Table 6:** Multiple logistic regression models for predictors of adequate knowledge of risk factors and warning signs for CVDs among young and middle-aged adults in rural Morogoro, Tanzania (*N* = 3000)

Variable	Knowledge of risk factors		Knowledge of warning signs	
	AOR (95% CI)	***p***-value	AOR (95% CI)	***p***-value
Age (years)				
25–34	Reference		Reference	
35–44	1.17 (0.96–1.42)		1.22 (1.01–1.49)	
45–54	1.07 (0.85–1.33)	0.087	1.45 (1.16–1.80)	0.003
55–64	0.85 (0.66–1.09)		0.98 (0.76–1.26)	
Gender				
Male	Reference		Reference	
Female	0.69 (0.57–0.84)	< 0.001	0.92 (0.77–1.12)	0.421
Marital status				
Never married	Reference		Reference	
Married	1.19 (0.94–1.50)	0.079	1.16 (0.92–1.46)	0.145
Separated/Divorced/Widowed	0.96 (0.72–1.29)		0.96 (0.72–1.29)	
Education level				
No formal education	Reference		Reference	
Primary education	1.45 (1.23–1.87)		1.74 (1.35–2.24)	
Secondary education	2.10 (1.42–3.10)	0.002	2.32 (1.59–3.39)	< 0.001
College/University	2.22 (0.92–5.39)		2.67 (1.13–6.30)	
District of residence				
Kilombero	Reference		Reference	
Ulanga	1.28 (1.10–1.50)	0.002	1.17 (1.00–1.37)	0.045
Occupation				
Farmer	Reference		Reference	
House wife	1.23 (0.75–2.01)		1.04 (0.64–1.67)	
Employed	0.92 (0.34–2.45)	0.519	1.03 (0.40–2.65)	0.170
Business	1.51 (0.89–2.58)		2.00 (1.16–3.45)	
Other	0.85 (0.42–1.69)		0.88 (0.44–1.75)	
Ever checked BP				
Yes	Reference		Reference	
No	0.55 (0.47–0.66)	< 0.001	0.51 (0.43–0.60)	< 0.001
Ever checked DM				
Yes	Reference		Reference	
No	0.59 (0.44–0.78)	< 0.001	0.48 (0.36–0.64)	< 0.001
Overweight/obese				
No	Reference		Reference	
Yes	1.29 (1.10–1.52)	0.002	1.18 (1.00–1.38)	0.042

### Self-perceived risk and practices towards CVD prevention

Nearly half (45.3%) of the participants perceived themselves to be at risk of cardiovascular disease. Although it was not statistically significant, women perceived themselves to be at risk of CVD more than men (75.7% vs 24.3%, *p* = 0.069). As for body weight, 30.7% perceived themselves to be overweight or obese. We found significant difference between actual body weight and perception about body weight where only about half (52.6%) of overweight and obese participants perceived their weight as overweight and obese (*p* < 0.001). Nearly one-third (31.9%) of the participants perceived that their body weight had increased in the past 1 year. With regard to practices, 5.7% of smokers had attempted to stop or decreased smoking while 21.7% had decreased drinking alcohol in the past 1 year. Other practices for prevention of CVD are shown in Table 7.

**Table 7:** **Perception and practices towards CVD prevention**

	Number (%)
**Self-perceived risk**	
Perceive to be at risk of cardiovascular disease	1360 (45.3)
Perceive to be overweight/obese	921 (30.7)
Perceive body weight has increased in the past 1 year	956 (31.9)
**Practices towards CVD prevention in the past 1 year**	
Conducted medical check-up for CVD risk factors	826 (27.5)
Decreased amount of food eaten so as loose or not gain weight	495 (16.5)
Decreased amount of salt in food	1412 (47.1)
Reduced consumption of fatty foods	1482 (49.4)
Decreased eating fast foods and out of home	1205 (40.2)
Exercise regularly	412 (13.7)
Attempted to stop /decreased smoking (among smokers)	10 (5.7)
Attempted to stop / decreased alcohol drinking (among drinkers)	128 (21.7)

## Discussion

To the best of our knowledge, the present study is the first to conduct comprehensive assessment of knowledge of risk factors and warning signs for CVDs and predictors of adequate knowledge scores among young and middle-aged adults in rural Tanzania.

### Source of information about CVDs

In our study, at least two-thirds of the participants had heard about CVDs. The main sources of knowledge of risk factors and warning signs for CVDs were relatives/neighbors, radio, other health care providers and doctors. Internet and social media were the least mentioned sources of information in this population mainly because of the rural nature of the study setting. It is also possible that people use internet and social media for other things rather than search for health information. We also observed sex differences in the sources of information with radio being the main source of information among men while relatives/neighbors were the main source among women. Other studies have also reported radio [22–24], relatives/neighbors [13, 38, 39] and health care providers [35, 40, 41] as sources of information for CVDs. Our findings have indicated underutilization of health care providers for provision of CVD-related health education. Deliberate efforts must be made to effectively use radio and health care providers as channels for delivery of health education programs to increase population knowledge of risk factors and warning signs in rural settings of Tanzania. A study in Kuwait demonstrated that higher knowledge of smoking, obesity, unhealthy diet and physical inactivity as CVD risk factors were attributable to their high mass media campaign [42].

### Knowledge of risk factors for CVDs

In our study, we found low level of knowledge of risk factors for CVDs among study participants with only quarter (25.4%) had good knowledge. Knowledge was much poor when we used open-ended questions as more than half of the participants (52.4%) could not mention even a single CVD risk factor. However, knowledge of risk factors improved when closed-ended questions were used and the proportion of participants who could not identify even a single CVD risk factor dropped to 6.9%. This improved knowledge with use of closed-ended questions may be due to most participants remembering risk factors when listed or guessed correct responses. More than half (51.4%) of the participants identified five or more risk factors. Similar to our findings, other community based studies have also reported low level of knowledge of risk factors for CVDs in the general population [13–15, 39]. On the contrary, high knowledge has been reported among studies that focused on special populations like health care providers [43, 44], secondary school teachers [23] and among staff of tertiary institution [45], meaning that education level and occupation have implication on health-related knowledge. Most participants in our study cited stress, obesity, cholesterol and physical inactivity as risk factors for CVDs. The proportion of participants who identified stress as a risk factor is comparable to that reported in a study conducted among Nigerian army force [24], but is much higher compare to other community based studies [14, 39, 46].

With exception of physical inactivity which was pointed out by nearly two thirds of the participants, smoking and alcohol were identified by only quarter of the participants as important risk factors for CVDs. A meta-analysis of 14 studies has shown smoking to be associated with up to 60% increased risk of stroke [47], and the risk was much higher for current smokers compared to ex-smokers. The literature also indicates a 30% increased risk for stroke among individual with second hand smoke exposure compared to those with no exposure at all [48]. Although the reported prevalence of current smoker in our study was low (5.9%), the national prevalence has previously been reported as 14.1% [49]. Thus, primary prevention interventions and particularly health education should be provided in order to increase population knowledge about risks associated with smoking and other modifiable risky behaviors like alcohol drinking and unhealthy diets.

### Knowledge of warning signs for CVD event

Only 17.5% of the participants had good knowledge of warning signs while more than half (55.2%) were not able to mention even a single warning sign for CVD event. Participants’ knowledge of warning signs was lower than knowledge for risk factors. Poor knowledge of warning signs for CVD event found in this study is similar to what has previously been reported in East Africa. A study in Uganda found that 57% of the participants did not know stroke warning signs or symptoms [15], whereas a study in Kenya found poor knowledge of warning signs for coronary heart disease among people living with HIV [22].

Shortness of breath was the most commonly identified warning sign for CVD event in our study. Less than half (44.1%) of participants identified chest pain as a warning sign for CVD event. These findings contrast results of the study that reported chest pain as the most commonly identified symptom [26]. Individuals with good knowledge of warning signs for CVD event are more likely to seek medical treatment early and consequently have better disease outcomes [50]. Additionally, people with good knowledge of both risk factors and warning signs tend to perceive themselves to be at higher risk for the disease and are more likely to adopt healthier lifestyle behaviors [51]. Thus, improving population knowledge of risk factors and warning signs for CVDs are likely to help adoption of healthier lifestyles for primary prevention, improve early medical care seeking behavior and consequently better prognosis among Tanzanians.

### Factors associated with CVD knowledge

In our study, socio-demographic and economic characteristics were associated with knowledge of risk factors and warning signs for CVDs. Knowledge of both risk factors and warning signs was higher among men compared to women. Our findings of higher CVD knowledge among men differs from other studies which reported higher CVD knowledge among females [42, 52]. Nonetheless, several other studies have reported insignificant gender differences in CVD knowledge [15, 53–55]. Higher CVD knowledge among women has been attributed to less working time among women which gives them more leisure time to watch and listen to mass media such as radio, television and newspaper [42]. In contrary, our study had found men to be listening, watching and reading from mass media than women which may explain higher CVD knowledge among men. More men than women reported radio, television and newspapers to be their main sources of information about CVDs.

The current study showed that participants aged 45–54 years were more knowledgeable about risk factors and warning signs compared to other age groups. These findings are suggestive that middle aged adults are more likely to seek health-related information than young individuals. Influence of age on CVD knowledge has also been reported by other studies conducted in SSA [39, 45, 56]. As expected, participants with higher education level and with formal employment were more knowledgeable about risk factors and warning signs compared to their counterparts. Other studies have also reported a positive association between higher education level with good CVD knowledge [12, 42, 54, 56, 57]. Educated individuals have higher capabilities to comprehend information including health messages delivered through various mass media channels.

Studies have reported geographical differences in CVD knowledge with those in urban settings having better knowledge compared to their counterparts in rural settings [16, 56]. Despite the fact that both Kilombero and Ulanga are rural districts, residents of Ulanga district had significantly higher knowledge about risk factors and warning signs than their counterparts from Kilombero district. Although this study did not assess ownership of community health fund (CHF) cards to draw its association with CVD knowledge, higher coverage of CHF overcomes the financial barrier to accessing health care services and consequently increase their likelihood of getting health-related messages and increase knowledge. Nonetheless, differences in CVD knowledge between the two rural districts warranties further exploration.

Behavior change is mostly influenced by one’s perception and knowledge that such behavior may cause harm to their health [58]. More than half (59.1%) of the participants had moderate to good knowledge of risk factors and warning signs for CVD. Consistent with knowledge, a reasonably good number (45.3%) of the participants perceived themselves to be at risk for CVD. Nearly one-third perceived themselves to be overweight or obese and that they had gained weight in the past 1 year. As for practices, only 5.7% of smokers had attempted to stop or decreased smoking while 21.7% had decreased drinking alcohol in the past 1 year. These findings indicate that interventions to increase population knowledge are needed to improved people’s perceptions and behavior change towards healthy lifestyle in order to combat the rising burden of CVDs in Tanzania.

## Conclusion

Knowledge of risk factors and warning signs in this rural population of young and middle-aged Tanzanian adults was generally still low. In order to combat the increasing burden of CVD risk factors in Tanzania, it is critical to develop and implement interventions that will improve population knowledge of risk factors. In this study, health care providers were not identified as key sources of information and knowledge about CVDs to the community, indicating that their primary focus is centered on treatment and that provision of preventive health messages is not emphasized. There is a need for paradigm shift to maximize the potential of health care providers as channels of health information to increase public knowledge of risk factors and warning signs especially in rural areas. As it is the case of health talk to pregnant women at the Reproductive and Child Health (RCH) clinics, such strategy can be translated so that outpatient clinics provide health talk on CVDs, risk factors and warning signs. With increased access to electricity through Rural Energy Agency (REA), cardiovascular-related health messages can be provided through Video Compact Discs (VCDs) played at outpatient clinics and visitors waiting areas. Additionally, community-based interventions through outreach programs by health care providers and CHWs as well as mass media campaigns should be implemented to improve public knowledge about CVDs and their risk factors. This way, rural communities will be empowered with knowledge to make informed choices regarding healthy lifestyles.

## Supplementary Information

**Additional file 1.**

## Data Availability

Datasets analysed for the current study are available from the corresponding author at reasonable request.

## Abbreviations

BMI: Body mass index
CHF: Community health fund
CHWs: Community health workers
CVDs: Cardiovascular diseases
DALYs: Disability adjusted life-years
HDSS: Health and demographic surveillance system
LMICs: Low and middle-income countries
RCH: Reproductive and child health
REA: Rural energy agency
SSA: Sub-Saharan Africa
VCDs: Video compact discs
WHO: World health organization
