# Supplementary material for: Public knowledge of risk factors and warning signs for cardiovascular disease among young and middle-aged adults in rural Tanzania
Source: BMC Public Health. 2020 Nov 30;20:1832. doi: 10.1186/s12889-020-09956-z (PMC7708242; doi:10.1186/s12889-020-09956-z)
Supplement: Supplementary file 1 — Additional file 1. [file 12889_2020_9956_MOESM1_ESM.docx]

MUHIMBILI UNIVERSITY OF HEALTH AND ALLIED SCIENCES


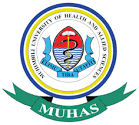


**Effects of Training Community Health Workers and Their Interventions on Cardiovascular Disease Risk Factors in Morogoro, Tanzania**

| **Basic Survey Information** |
| --- |

| Location and Date | Response | | Code |
| --- | --- | --- | --- |
| District | Kilombero = 1 | Ulanga = 2 |  |
| Village | LIP = 1 MAN = 4 KIV = 7 LUP = 10  MLA = 2 SON = 5 MIN = 8 KIC = 11  LUM = 3 SOL = 6 IGU = 9 IGO = 12 | |  |
| Hamlet | ......................................................................... | |  |
| Ten Cell Leader Name | ......................................................................... | |  |
| Date of interview | └─┴─┘ └─┴─┘ └─┴─┴─┴─┘  dd mm year | |  |
| Phone number | ………………………………………………………. | |  |

| **Demographic Information** | | | | | |
| --- | --- | --- | --- | --- | --- |
|  | **Demographic Information** | | | |  |
|  | **Question** | **Response** | | **Code** |  |
| *1* | Participant’s sex | Male = 1 | Female = 2 |  |  |
| *2* | What is your date of birth?  *DK= 88 88 8888* | └─┴─┘ └─┴─┘ └─┴─┴─┴─┘  dd mm year | |  |  |
| 3 | How old are you? | Years | └─┴─┘ |  |  |
| 4 | What is the **highest level of education** you have completed? | No formal schooling  Less than primary school  Primary school completed  Ordinary secondary completed  Advanced secondary school completed  College/University completed  Postgraduate degree  Refused | = 1  = 2  = 3  = 4  = 5  = 6  = 7  = 88 |  |  |
| 5 | In total, how many years have you spent at school (excluding pre-school)? | Years | └─┴─┘ |  |  |
| 6 | What is your **marital status**? | Single  Currently married  Separated  Divorced  Widowed  Cohabiting  Refused | = 1  = 2  = 3  = 4  = 5  = 6  = 88 |  |  |
| 7 | Which of the following best describes your **main** **work** status over the past 12 months? | Farmer  Housewife  Employed  Business/Petty trade  Student  Retired  None  Refused | = 1  = 2  = 3  = 4  = 5  = 6  = 7  = 88 |  |  |
| 8 | How many people **older than 18** years, including yourself, live in your household? | Children (<5 years) _________  Children (5-17 years) _________  Adults (≥18 years) ________ | |  |  |
| 9 | The walls of the house you live in, what are they made of? | Mud = 1  Raw bricks = 2  Mud and plaster = 3  Bricks = 4  Bricks and plaster = 5  Block bricks = 6 |  |  |  |
| 10 | What materials are used for **house roofing**? | Grass  Iron sheets  Roofing tiles  concrete  Other | = 1  = 2  = 3  = 4  = 5 |  |  |
| 11 | What materials are used for the **house floor**? | None  Concrete/Cement  Floor tiles  Wood  Other | = 1  = 2  = 3  = 4  = 5 |  |  |
| 12 | Number of sleeping rooms | Number | _____ |  |  |
| 13 | *Does your household own any of the following?* | | | |  |
| 13.1 | Electricity (working) | Yes = 1 No = 2 | DK = 88 |  |  |
| 13.2 | Generator (working) | Yes = 1 No = 2 | DK = 88 |  |  |
| 13.3 | Tape water | Yes = 1 No = 2 | DK = 88 |  |  |
| 13.4 | Coach or chair | Yes = 1 No = 2 | DK = 88 |  |  |
| 13.5 | Television | Yes = 1 No = 2 | DK = 88 |  |  |
| 13.6 | Radio | Yes = 1 No = 2 | DK = 88 |  |  |
| 13.7 | Refrigerator | Yes = 1 No = 2 | DK = 88 |  |  |
| 13.8 | Fan | Yes = 1 No = 2 | DK = 88 |  |  |
| 13.9 | Bicycle | Yes = 1 No = 2 | DK = 88 |  |  |
| 13.10 | Motorcycle | Yes = 1 No = 2 | DK = 88 |  |  |
| 13.11 | Motor vehicle | Yes = 1 No = 2 | DK = 88 |  |  |
| 14 | What type of fuel does your household mainly use for cooking? | Firewood  Charcoal  Kerosene  Electricity/Natural gas  Biogas  Dung  Other  Don’t know | = 1  = 2  = 3  = 4  = 5  = 6  = 7  = 88 |  |  |
| 15 | What is the main source of drinking water for your household? | Bottled water  Piped/Tape water at home  Public tape water  Tube well/Bore hole  Protected well  Unprotected well  River/Ponds  Don’t know | = 1  = 2  = 3  = 4  = 5  = 6  = 7  = 88 |  |  |
| 16 | What type of toilet does your household use | None =1  Pit toilet = 2  Flash toilet = 3 |  |  |  |
| 17 | How many of the following livestock are owned by your household | Cow ___________ Goat _________  Sheep _________ Chicken ________ |  |  |  |

| **Knowledge about CVD** | | | | | |
| --- | --- | --- | --- | --- | --- |
|  | **Knowledge about CVDs** | | | |  |
|  | *Now I am going to ask you some questions related to cardiovascular diseases* | | | |  |
|  | **Question** | **Response** | | **Code** |  |
| 18 | Have you ever heard about cardiovascular diseases? | Yes = 1 | No = 2 |  |  |
| 19 | If yes, where did you hear from?  *(CHECK ALL THAT APPY)* | Radio  Television  Newspaper/Magazine  Doctor  Other health worker  Relatives/Neighbors  Internet  Other sources  N/A | = 1  = 2  = 3  = 4  = 5  = 6  = 7  = 8  = 99 |  |  |
|  | *Knowledge about risk factors for CVDs* | | | |  |
| 20 | Do you know the risk factors for cardiovascular diseases? | Yes = 1 | No = 2 |  |  |
| 21 | If yes, can you mention the risk factors for cardiovascular diseases?  *(LET THE PARTICIPANT MENTION, AND CIRCLE ALL THAT APPY)* | Advanced/Older age  Overweight/Obesity  hypertension  Diabetes mellitus  Dyslipidemia  Smoking  Excessive alcohol consumption  Physical inactivity  Family history  Stress  Don’t know | = 1  = 2  = 3  = 4  = 5  = 6  = 7  = 8  = 9  = 10  = 88 |  |  |
| 22 | How many cardiovascular disease risk factors have been mentioned by participant?  *DK = 88* | Number | └─┴─┘ |  |  |
| 23 | Which of the following are risk factors for cardiovascular diseases?  *(READ OUT TO THE CLIENT AND RECORD FOR EACH)* | | | |  |
| 23.1 | Older age | Yes = 1 No = 2 | DK = 88 |  |  |
| 23.2 | Obesity/Overweight | Yes = 1 No = 2 | DK = 88 |  |  |
| 23.3 | Hypertension | Yes = 1 No = 2 | DK = 88 |  |  |
| 23.4 | Diabetes mellitus | Yes = 1 No = 2 | DK = 88 |  |  |
| 23.5 | Dyslipidemia | Yes = 1 No = 2 | DK = 88 |  |  |
| 23.6 | Smoking | Yes = 1 No = 2 | DK = 88 |  |  |
| 23.7 | Excessive alcohol consumption | Yes = 1 No = 2 | DK = 88 |  |  |
| 23.8 | Physical inactivity | Yes = 1 No = 2 | DK = 88 |  |  |
| 23.9 | History of heart disease | Yes = 1 No = 2 | DK = 88 |  |  |
| 23.10 | Family history | Yes = 1 No = 2 | DK = 88 |  |  |
| 23.11 | Stress | Yes = 1 No = 2 | DK = 88 |  |  |
| 23.12 | Kidney disease | Yes = 1 No = 2 | DK = 88 |  |  |
|  | *Knowledge about warning signs for cardiovascular disease* | | | |  |
| 24 | Do you know the warning signs for cardiovascular disease event | Yes = 1 | No = 2 |  |  |
| 25 | If yes, can you mention the warning signs for cardiovascular disease event?  *(LET THE PARTICIPANT MENTION, AND CIRCLE ALL THAT APPY)* | Headache  Chest pain  Dyspnea  Sweating  Vomiting  Pain in the teeth/jaw  Pain/numbness in the arm  Loss of consciousness  Dizziness or light headedness  Don’t know | = 1  = 2  = 3  = 4  = 5  = 6  = 7  = 8  = 9  = 88 |  |  |
| 26 | How many warning signs have been mentioned by the participant?  *DK = 88* | Number | └─┴─┘ |  |  |
| 27 | Which of the following are warning signs for cardiovascular diseases event?  *(READ OUT TO THE CLIENT AND RECORD FOR EACH)* | | | |  |
| 27.1 | Headache | Yes = 1 No = 2 | DK = 88 |  |  |
| 27.2 | Chest pain | Yes = 1 No = 2 | DK = 88 |  |  |
| 27.3 | Dyspnea | Yes = 1 No = 2 | DK = 88 |  |  |
| 27.4 | Sweating | Yes = 1 No = 2 | DK = 88 |  |  |
| 27.5 | Vomiting | Yes = 1 No = 2 | DK = 88 |  |  |
| 27.6 | Pain in the teeth or jaw | Yes = 1 No = 2 | DK = 88 |  |  |
| 27.7 | Pain or numbness in the arm | Yes = 1 No = 2 | DK = 88 |  |  |
| 27.8 | Loss of consciousness | Yes = 1 No = 2 | DK = 88 |  |  |
| 27.9 | Dizziness or light headedness | Yes = 1 No = 2 | DK = 88 |  |  |
|  | Knowledge about course of action in case of cardiovascular disease event | | | |  |
| 28 | Which of the following will you do in case of a cardiovascular diseases event?  *(READ OUT TO THE CLIENT AND RECORD FOR EACH)* | | | |  |
| 28.1 | Immediately take the person to hospital | Yes = 1 | No = 2 |  |  |
| 28.2 | Immediately take the person to drug store | Yes = 1 | No = 2 |  |  |
| 28.3 | Treat the person at home/ Give home-made therapy | Yes = 1 | No = 2 |  |  |
| 28.4 | Take the person to traditional healer | Yes = 1 | No = 2 |  |  |
| 28.5 | Wait for appointment to hospital/clinic | Yes = 1 | No = 2 |  |  |
| 28.6 | Wait for condition to subside on its own | Yes = 1 | No = 2 |  |  |
| 28.7 | Other | Yes = 1 | No = 2 |  |  |
| 28.8 | Don’t know | Yes = 1 | No = 2 |  |  |
|  | Knowledge about healthy dietary habits for cardiovascular disease | | | |  |
| 29 | Which of the following foods are good/healthy for prevention of cardiovascular diseases  *(READ OUT TO THE CLIENT AND RECORD FOR EACH)* | | | |  |
| 29.1 | Green leafy vegetables | Yes = 1 No = 2 | DK = 88 |  |  |
| 29.2 | Fruits | Yes = 1 No = 2 | DK = 88 |  |  |
| 29.3 | Salty foods | Yes = 1 No = 2 | DK = 88 |  |  |
| 29.4 | Fast foods | Yes = 1 No = 2 | DK = 88 |  |  |
| 29.5 | Whole grain foods | Yes = 1 No = 2 | DK = 88 |  |  |
| 29.6 | High fat diet | Yes = 1 No = 2 | DK = 88 |  |  |

| **Medical history** | | | | | |
| --- | --- | --- | --- | --- | --- |
|  | **General Health** | | | |  |
|  | *I will now ask you about questions related to your health* | | | |  |
|  | **Question** | **Response** | | **Code** |  |
| 30 | In general, how do you rate your health **today**? | Good  Moderate  Bad  Refused/Don’t know | = 1  = 2  = 3  = 88 |  |  |
| 31 | In general, how do you rate your health for the **past one month**? | Good  Moderate  Bad  Refused/Don’t know | = 1  = 2  = 3  = 88 |  |  |
| 32 | In case you fall sick, where do you **go first** for medical care? | Dispensary  Health centre  Hospital  Essential drug store  Traditional healer  Other  Refused/Don’t know | = 1  = 2  = 3  = 4  = 5  = 6  = 88 |  |  |
|  | History of Raised Blood Pressure (Hypertension) | | | |  |
| 33 | Have you ever had your blood pressure measured by a doctor or other health worker? | Yes =1 | No = 2 |  |  |
| 34 | Have you ever been told by a doctor or other health worker that you have raised blood pressure or hypertension? | Yes = 1 No = 2 | N/A = 99 |  |  |
| 35 | Are you currently taking any drugs (medication) for raised blood pressure prescribed by a doctor or other health worker? | Yes = 1 No = 2 | N/A = 99 |  |  |
| 36 | Have you ever seen a traditional healer for raised blood pressure or hypertension? | Yes = 1 No = 2 | N/A = 99 |  |  |
| 37 | Are you currently taking any herbal or traditional remedy for your raised blood pressure? | Yes = 1 No = 2 | N/A = 99 |  |  |
|  | History of Raised Blood Sugar (Diabetes Mellitus) | | | |  |
| 38 | Have you ever had your blood sugar measured by a doctor or other health worker? | Yes =1 | No = 2 |  |  |
| 39 | Have you ever been told by a doctor or other health worker that you have raised blood sugar or diabetes? | Yes = 1 No = 2 | N/A = 99 |  |  |
| 40 | Are you currently taking any drugs (medication) for diabetes prescribed by a doctor or other health worker? | Yes = 1 No = 2 | N/A = 99 |  |  |
| 41 | If yes, what medications are you taking? | Oral drugs = 1 Insulin = 2 | NA =99 |  |  |
| 42 | Have you ever seen a traditional healer for raised blood sugar or diabetes? | Yes = 1 No = 2 | N/A = 99 |  |  |
| 43 | Are you currently taking any herbal or traditional remedy for your diabetes? | Yes = 1 No = 2 | N/A = 99 |  |  |
|  | History of Raised Blood Lipids (Dyslipidemia) | | | |  |
| 44 | Have you ever had your cholesterol (fat levels in your blood) measured by a doctor or other health worker? | Yes =1 | No = 2 |  |  |
| 45 | Have you ever been told by a doctor or other health worker that you have raised cholesterol? | Yes = 1 No = 2 | N/A = 99 |  |  |
| 46 | Are you currently taking any oral treatment (medication) for raised total cholesterol prescribed by a doctor or other health worker? | Yes = 1 No = 2 | N/A = 99 |  |  |
| 47 | Have you ever seen a traditional healer for raised cholesterol? | Yes = 1 No = 2 | N/A = 99 |  |  |
| 48 | Are you currently taking any herbal or traditional remedy for your raised cholesterol? | Yes = 1 No = 2 | N/A = 99 |  |  |
|  | History of Cardiovascular Disease | | | |  |
| 49 | Have you ever been told by a doctor or other health worker that you have heart disease? | Yes =1 | No = 2 |  |  |
| 50 | Have you ever had a heart attack or chest pain from heart disease (angina) or a stroke (cerebrovascular accident or incident)? | Yes =1 | No = 2 |  |  |
| 51 | Are you currently taking aspirin regularly to prevent or treat heart disease? | Yes = 1 No = 2 | N/A = 99 |  |  |
| 52 | Are you currently taking any other medications regularly to prevent or treat heart disease? | Yes = 1 No = 2 | N/A = 99 |  |  |
| 53 | Have you ever seen a traditional healer for cardiovascular disease? | Yes = 1 No = 2 | N/A = 99 |  |  |
| 54 | Are you currently taking any herbal or traditional remedy for cardiovascular disease? | Yes = 1 No = 2 | N/A = 99 |  |  |
|  | Lifestyle Advice | | | |  |
| 55 | During the past three years, has a doctor or other health worker advised you to do any of the following?  (ASK AND RECORD FOR EACH) | | | |  |
| 55.1 | Quit using tobacco or don’t start | Yes =1 | No = 2 |  |  |
| 55.2 | Reduce salt in your diet | Yes =1 | No = 2 |  |  |
| 55.3 | Eat at least five servings of fruit and/or vegetables each day | Yes =1 | No = 2 |  |  |
| 55.4 | Reduce fat in your diet | Yes =1 | No = 2 |  |  |
| 55.5 | Start or do more physical activity | Yes =1 | No = 2 |  |  |
| 55.6 | Maintain a healthy body weight or lose weight | Yes =1 | No = 2 |  |  |

| **Behavior Towards CVD** | | | | |
| --- | --- | --- | --- | --- |
|  | **Tobacco Use** | | | |
|  | *Now I am going to ask you some questions about tobacco use* | | | |
|  | **Question** | **Response** | | **Code** |
| 56 | Currently, do you smoke any **tobacco** products, such as cigarettes, cigars or pipes? | Yes = 1 | No = 2 |  |
| 57 | If no, have you ever smoked tobacco products | Yes = 1 No = 2 | N/A = 99 |  |
| 58 | How old were you when you **started** smoking tobacco products?  *N/A=99* | Age (years) | └─┴─┘ |  |
| 59 | *(For current smoker)*  On average, **how many** of the following tobacco products do you smoke **each day/week?**  *DK = 88 N/A = 99* | **DAILY↓** | **WEEKLY↓** |  |
|  |  | Manufactured cigarettes └─┴─┘ | └─┴─┘ |  |
|  |  | Hand-rolled cigarettes └─┴─┘ | └─┴─┘ |  |
| 60 | During the past 12 months, have you tried to **stop smoking**? | Yes = 1 No = 2 | N/A = 99 |  |
| 61 | Do you **currently use** any **smokeless tobacco** products such as *[snuff, chewing tobacco]*? | Yes = 1 | No = 2 |  |
| 62 | On average, how many **times a day or per week** do you use smokeless tobacco products | **DAILY↓** | **WEEKLY↓** |  |
|  |  | └─┴─┘ | └─┴─┘ |  |
|  | **Alcohol consumption** | | | |
|  | *The following questions ask about consumption of alcohol* | | | |
| 63 | Currently, do you drink any alcohol such as beer, wine, spirits or local brews | Yes = 1 | No = 2 |  |
| 64 | If no, have you **ever** consumed any alcohol such as beer, wine, spirits or local brews in the past? | Yes = 1 | No = 2 |  |
| 65 | Have you stopped drinking due to health reasons, such as a negative impact on your health or on the advice of your doctor or other health worker? | Yes = 1 No = 2 | N/A = 99 |  |
| 66 | Have you consumed any alcohol in the **past 30 days**? | Yes = 1 No =2 | N/A = 99 |  |
| 67 | During the **past 30 days**, on how many **occasions** did you have at least one standard alcoholic drink? | Daily  4-6 Days per week  1-3 days per week  1-3 days per month  N/A | = 1  = 2  = 3  = 4  = 99 |  |
| 68 | On average, during each of the **past 7 days**, on how many **occasions** did you have at least one standard alcoholic drink? | Daily  4-6 Days per week  1-3 days per week  N/A | = 1  = 2  = 3  = 99 |  |
|  | **Diet (Fruits and Vegetables)** | | | |
|  | *The next questions ask about the fruits and vegetables that you usually eat. I have a nutrition card here that shows you some examples of local fruits and vegetables. Each picture represents the size of a serving. As you answer these questions please think of a typical week in the last year.* | | | |
| 69 | In a typical week, on how many days do you **eat fruit**?  *DK = 88* | Number of days | └─┴─┘ |  |
| 70 | In a typical week, on how many days do you **eat vegetables**?  *DK = 88* | Number of days | └─┴─┘ |  |
|  | **Dietary Salt** | | | |
|  | With the next questions, we would like to learn more about salt in your diet. Dietary salt includes ordinary table salt, unrefined salt such as sea salt, iodized salt and powders. The following questions are on how food is prepared in your home, and on adding salt to the food right before you eat it. Please answer the questions even if you consider yourself to eat a diet low in salt. | | | |
| 71 | How often is **salt added** in cooking or preparing foods in your household? | Always  Often  Sometimes  Never | = 1  = 2  = 3  = 4 |  |
| 72 | How often do you **add salt** to your food right before you eat it or as you are eating it? | Always  Often  Sometimes  Never | = 1  = 2  = 3  = 4 |  |
| 73 | **How much salt** do you think you consume? | Too much  Just the right amount  Too little  DK | = 1  = 2  = 3  = 88 |  |
| 74 | Do you think that too much salt in your diet could cause any **health problems**? | Yes = 1 No = 2 | DK = 88 |  |

| **Perceptions and Attitudes Towards CVD** | | | | | |
| --- | --- | --- | --- | --- | --- |
|  | **Perception about cardiovascular disease risk** | | | |  |
|  | *Now, I will ask you questions about your perception and attitudes towards cardiovascular diseases* | | | |  |
|  | **Question** | **Response** | | **Code** |  |
| 75 | Do you perceive yourself to be at risk of cardiovascular disease? | Yes = 1 | No = 2 |  |  |
| 76 | How do you perceive your current body weight? | Underweight  Normal  Overweight  Obese | = 1  = 2  = 3  = 4 |  |  |
| 77 | In the past one year, has your weight changed? | Decreased  Not changed  Increased | = 1  = 2  = 3 |  |  |

| **Practices Towards Cardiovascular Diseases** | | | | | |
| --- | --- | --- | --- | --- | --- |
|  | ***Finally, I am going to ask you about things you have done to prevent yourself from cardiovascular diseases.*** | | | |  |
|  | *In the past one year, which of the following have you done to help you have better heart health* | | | |  |
|  | **Question** | **Response** | | **Code** |  |
|  | **General practices** | | | |  |
| 78 | Reduce or maintain healthy weight | Yes = 1 | No = 2 |  |  |
| 79 | Increase knowledge about cardiovascular disease and risk factors | Yes = 1 | No = 2 |  |  |
| 80 | Medical checkup for cardiovascular disease risk factors | Yes = 1 | No = 2 |  |  |
|  | **Practices towards diet** |  |  |  |  |
| 81 | Limit amount of food you eat in order to lose or not to gain weight | Yes = 1 | No = 2 |  |  |
| 82 | Decreased the amount of salt in the diet | Yes = 1 | No = 2 |  |  |
| 83 | Reduced fatty foods | Yes = 1 | No = 2 |  |  |
| 84 | Decrease eating fast foods and out of home | Yes = 1 | No = 2 |  |  |
|  | **Practices towards physical activity** | | | |  |
| 85 | Exercise regularly | Yes = 1 | No = 2 |  |  |
|  | **Practices towards cardiovascular disease risk behaviors** | | | |  |
| 86 | Stopped smoking | Yes = 1 No = 2 | N/A = 99 |  |  |
| 87 | Stopped drinking or decreased the amount of alcohol I consume | Yes = 1 No = 2 | N/A = 99 |  |  |

| **Physical and Biochemical Measurements** | | | | |
| --- | --- | --- | --- | --- |
|  | **Anthropometric Measurements** | | | |
|  | **Question** | **Response** | | **Code** |
| 88 | Height | └─┴─┴─┘. └─┘ | (cm) |  |
| 89 | Weight | └─┴─┴─┘. └─┘ | (kg) |  |
| 90 | Waist circumference | └─┴─┴─┘. └─┘ | (cm) |  |
| 91 | Hip circumference | └─┴─┴─┘. └─┘ | (cm) |  |
|  | **Blood Pressure Measurements** | | | |
| 92.1 | Blood pressure reading 1 | Systolic └─┴─┴─┘ | (mmHg) |  |
|  |  | Diastolic └─┴─┴─┘ | (mmHg) |  |
|  |  | Heart rate └─┴─┴─┘ | (beats/min) |  |
| 92.2 | Blood pressure reading 2 | Systolic └─┴─┴─┘ | (mmHg) |  |
|  |  | Diastolic └─┴─┴─┘ | (mmHg) |  |
|  |  | Heart rate └─┴─┴─┘ | (beats/min) |  |
| 92.3 | Blood pressure reading 3 | Systolic └─┴─┴─┘ | (mmHg) |  |
|  |  | Diastolic └─┴─┴─┘ | (mmHg) |  |
|  |  | Heart rate └─┴─┴─┘ | (beats/min) |  |

**THANK YOU FOR YOUR PARTICIPATION**
